# Population Structure of the Chagas Disease Vector *Triatoma infestans* in an Urban Environment

**DOI:** 10.1371/journal.pntd.0003425

**Published:** 2015-02-03

**Authors:** Camilo E. Khatchikian, Erica A. Foley, Corentin M. Barbu, Josephine Hwang, Jenny Ancca-Juárez, Katty Borrini-Mayori, Victor R. Quıspe-Machaca, Cesar Naquira, Dustin Brisson, Michael Z. Levy, The Chagas Disease Working Group in Arequipa

**Affiliations:** 1 Department of Biology, University of Pennsylvania, Philadelphia, Pennsylvania, United States of America; 2 Department of Biostatistics and Epidemiology, University of Pennsylvania Perelman School of Medicine, Philadelphia, Pennsylvania, United States of America; 3 Facultad de Ciencias y Filosofía, Universidad Peruana Cayetano Heredia, Lima, Peru; Liverpool School of Tropical Medicine, UNITED KINGDOM

## Abstract

Chagas disease is a vector-borne disease endemic in Latin America. *Triatoma infestans*, a common vector of this disease, has recently expanded its range into rapidly developing cities of Latin America. We aim to identify the environmental features that affect the colonization and dispersal of *T. infestans* in an urban environment. We amplified 13 commonly used microsatellites from 180 *T. infestans* samples collected from a sampled transect in the city of Arequipa, Peru, in 2007 and 2011. We assessed the clustering of subpopulations and the effect of distance, sampling year, and city block location on genetic distance among pairs of insects. Despite evidence of genetic similarity, the majority of city blocks are characterized by one dominant insect genotype, suggesting the existence of barriers to dispersal. Our analyses show that streets represent an important barrier to the colonization and dispersion of *T. infestans* in Arequipa. The genetic data describe a *T. infestans* infestation history characterized by persistent local dispersal and occasional long-distance migration events that partially parallels the history of urban development.

## Author Summary

The colonization and dispersal of disease vectors in new and expanding urban areas pose important health risks. The population and demographic dynamics of these vectors are often unclear, and their temporal and spatial associations with urbanization are unknown. Here, we use molecular markers to describe the genetic structure of populations of *T. infestans*, an important vector of the etiologic agent of Chagas disease, in an expanding urban environment. Samples were obtained along a transect in Arequipa, Peru, that includes old, well-developed communities and new communities characterized by rudimentarily constructed houses. We assessed the clustering of subpopulations and the effect of distance, sampling year, and city block on genetic distance among pairs of insects. Our analyses show that streets represent an important barrier to the colonization and dispersion of *T. infestans* in Arequipa. The genetic data describe a *T. infestans* infestation history characterized by persistent local dispersal and occasional long-distance migration events that partially parallels the history of urban development.

## Introduction

Chagas disease is a vector-borne disease endemic in Latin America that poses important public health risks [1]. *Triatoma infestans*, a true bug that commonly harbors the etiologic agent of Chagas disease, *Trypanosoma cruzi*, has historically occurred throughout southern South America [2,3]. Its range has expanded from sparsely populated rural areas into densely populated urban areas, and it often lives within the walls of rudimentarily built houses [4–6]. Rapid urbanization results in proximity between humans and pest species, including rodents and insects, many of which carry pathogens that are transmittable to humans. The rate at which these pest species disperse through human communities is closely associated with the disease risk of the human population [7] and with economic costs [8].

Urban landscapes are a patchwork of habitats composed of a heterogeneous mosaic of city blocks separated by inhospitable streets [9]. Features of urban landscapes provide unique challenges and opportunities for a species to colonize and proliferate. Identifying features that promote or hinder colonization and migration in cities enables a mechanistic understanding of the distribution and abundance of organisms in urban environments [10,11]. Furthermore, the recognition of these environmental barriers is important for designing effective control measures. Studies assessing the impact of anthropogenic landscape alterations on species still living in the remnants of their unaltered habitat are common. In contrast, few studies have examined how species colonize and disperse throughout the urban landscape itself. This bias is surprising given the proximity between humans and these pest species and the economic, ecological, social, and public health implications of infestations [8]. Here we use molecular methods to elucidate the environmental features of a growing urban landscape that affect the colonization and dispersal of *T. infestans* along a west-to-east transect within the city of Arequipa, Peru.

## Materials and Methods

### Study transect

**Fig 1:**
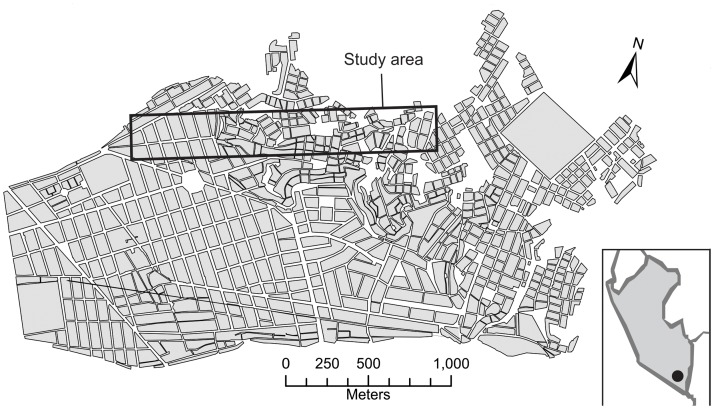
General map of the Mariano Melgar district of Arequipa, Peru; gray polygons represent city blocks. The transect area is outlined with a bold rectangle. Inset shows the location of the city of Arequipa in Peru.

The study transect is located in the Mariano Melgar district of Arequipa, Peru (Fig. 1). The transect follows a gradient from old, well-developed communities in the west near the city center to new communities in the east. The new communities are characterized by recent and rudimentarily constructed homes that extend to the current eastern border of the urbanized area. A lack of mortar between the blocks of volcanic stone and bricks that make up the walls of these rudimentarily built homes creates an ideal habitat for *T. infestans* [4].

### Sample collection and processing

The *T. infestans* samples used in this study were collected in 2008 and 2011 from homes in the transect area; Tetramethrin (Sapolio, Mata Moscas) was sprayed into the cracks in stone walls or other small structures that provide suitable *T. infestans* habitats to flush out the insects. More than 3,000 individuals were collected, placed into individual tubes, and stored at -20°C. From this total, 180 were chosen for genetic analyses (90 from each sampled year). To choose specific samples for analysis, 30 houses were randomly chosen from each third of the transect (equally divided along the length of the transect) and one fifth instar or adult individual was randomly selected from each chosen home.

Genomic DNA was extracted from two legs of each sample following the insect tissue protocol provided with the Qiagen DNEasy Blood and Tissue kit (Valencia, CA, USA). The 13 microsatellites used in this study are well characterized, commonly used for both macrogeographical and microgeographical studies [e.g., 6,12–14], and have been found to be in linkage equilibrium [15–17]. They were amplified using a fluorescent-tagged forward primer (ABI dyes: 6-FAM, PET, VIC, or NED) using standard protocols. Complete sequences, cycling conditions and source literature are described in S1 Table. Fragment sizing was completed at the DNA Sequencing Center (Applied Biosystems 3100 Capillary Sequencer and GeneMApper) of the University of Pennsylvania. Electropherograms were visualized in PeakScanner (ABI) to confirm the automated allele sizing. Ambiguous peaks were re-amplified and re-genotyped until clear allele sizes were obtained. Alleles were binned according to the established repeat size with TANDEM [18]. Negative controls were run with all PCR reactions to prevent cross-contamination and included in fragment sizing analyses.

### Analyses

Potential differences between collection years were assessed with the exact test of sample differentiation in Arlequin version 3.5.1.2 [19] using 100,000 Markov steps. Population genetic structure was assessed using the Bayesian clustering algorithm implemented by STRUCTURE version 2.3 [20–22]. We ran five independent iterations of the analysis for each number of genetic clusters (*K*, ranging from 2 to 12) assuming correlated allele frequencies, admixture, and no location data as a prior, with 500,000 Markov Chain Monte Carlo (MCMC) iterations and a 20% burn-in. The output of the STRUCTURE analyses was extracted in STRUCTURE HARVESTER [23] and the optimal alignment of the five iterations was determined using CLUMPP [24]. The *ΔK* method implemented by Structure Harvester was used to determine the optimal number of clusters [25]. The cluster assignment of each individual was plotted in geographic space in ArcMap 10 [26] as a pie chart. The individual alleles were plotted in a similar manner to verify the detected trends.

We used a permutation-based linear regression to quantify the effect of Euclidean distance, sampling year, and city block (same or different city block coded as 1 and 0, respectively) on the genetic distance between pairs of insects using the Brat-Curtis dissimilarity index [27]. Null distributions of the effect of each variable on genetic distance were constructed by randomly permuting genotypes among individuals collected in the same year, and the effect size of each variable on the genetic distance between pairs of individuals was calculated as the regression coefficient in a linear regression [28]. The regression coefficients calculated from the unpermuted (observed) data were compared to the null distribution of coefficients calculated from 10,000 permutations to derive a p-value for each parameter estimate. Violin plots were used to visualize the effect of environmental factors on genetic distance using R [27]. Additionally, a restricted data set consisting of matching equal-distance pairs of samples (with 1 m precision) located either in the same city block or in different blocks was analyzed in JMP version 10 [29]. This analysis allows a direct comparison of pairs found at the same spatial scale to determine the effect of streets on the fine-scale structure of the insect population.

Point estimates of population genetic diversity across the transect were calculated using sGD [28]. The analyses calculate the observed heterozygosity, the inbreeding coefficient (*F*_IS_), and the allelic richness around each individual sample in the defined radius considering all samples within that radius. Data points with fewer than 10 individuals within the radius were excluded from the analyses. The optimal distance was defined as the distance at which the number of valid samples levels off. Radii of 100, 125, 150, 175, 200, 225, and 250 m were considered. The indexes obtained with the selected radius were plotted using ArcMap 10. To assess the presence of trends across the transect, the calculated indexes (observed heterozygosity, inbreeding coefficient (*F*_IS_), and allelic richness) were regressed against the horizontal position in the transect using JMP 10.

### Data accessibility

Microsatellite data generated in this study have been deposited to DRYAD and are available from the Dryad Digital Repository: http://doi.org/10.5061/dryad.5tt50.

## Results

**Fig 2:**
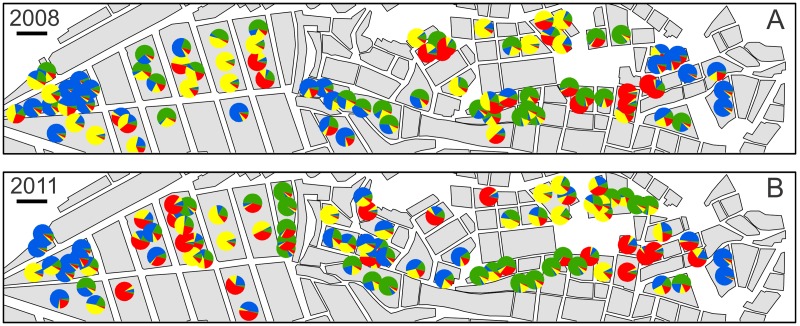
Results of the STRUCTURE genetic cluster analysis with four genetic clusters in 2008 (A) and 2011 (B). The colors of each pie chart represent the likelihood of the individual’s assignment to that cluster. The black bar represents 100 m. Data points have been relocated (jiggled) inside blocks to protect residents’ privacy.

Using the exact test of sample differentiation, we found no difference (p > 0.05) in genotype frequencies between the samples collected in 2008 and those collected in 2011. The genetic analyses showed that *T. infestans* populations are very finely spatially structured within the sampled transect. The majority of city blocks can be characterized by a single, dominant subpopulation as defined by the STRUCTURE algorithm, which incorporates information from all analyzed loci (Fig. 2). Samples belonging to the same block are assigned to the same subpopulation more frequently than expected by chance (56% observed vs. 27% expected; χ2 = 84.96, p < 0.001). The rapid changes in subpopulations from one city block to the next and the patchy distribution of these subpopulations are easily visualized by mapping the STRUCTURE subpopulation probability of each sample onto the study area. These patterns and the dominant subpopulation on each city block were consistent in both of the sampling periods (Fig. 2A, B). The optimal number of subpopulations is assumed to be four (S1 Fig.) by the ∆*K* method [25]; however.

The fine-scale spatial patterns are also visible for each of the 13 analyzed loci (S2 Fig.). In nearly all cases, a single allele from each locus dominates a city block; however, there are also intermediate zones where two alleles occupy the same block, resulting in heterozygous individuals (S2 Fig.). Furthermore, each allele is found throughout the transect, reflecting a similar pattern that was previously found with the STRUCTURE algorithm wherein each identified subpopulation was also found throughout the transect (Fig. 2).

**Fig 3:**
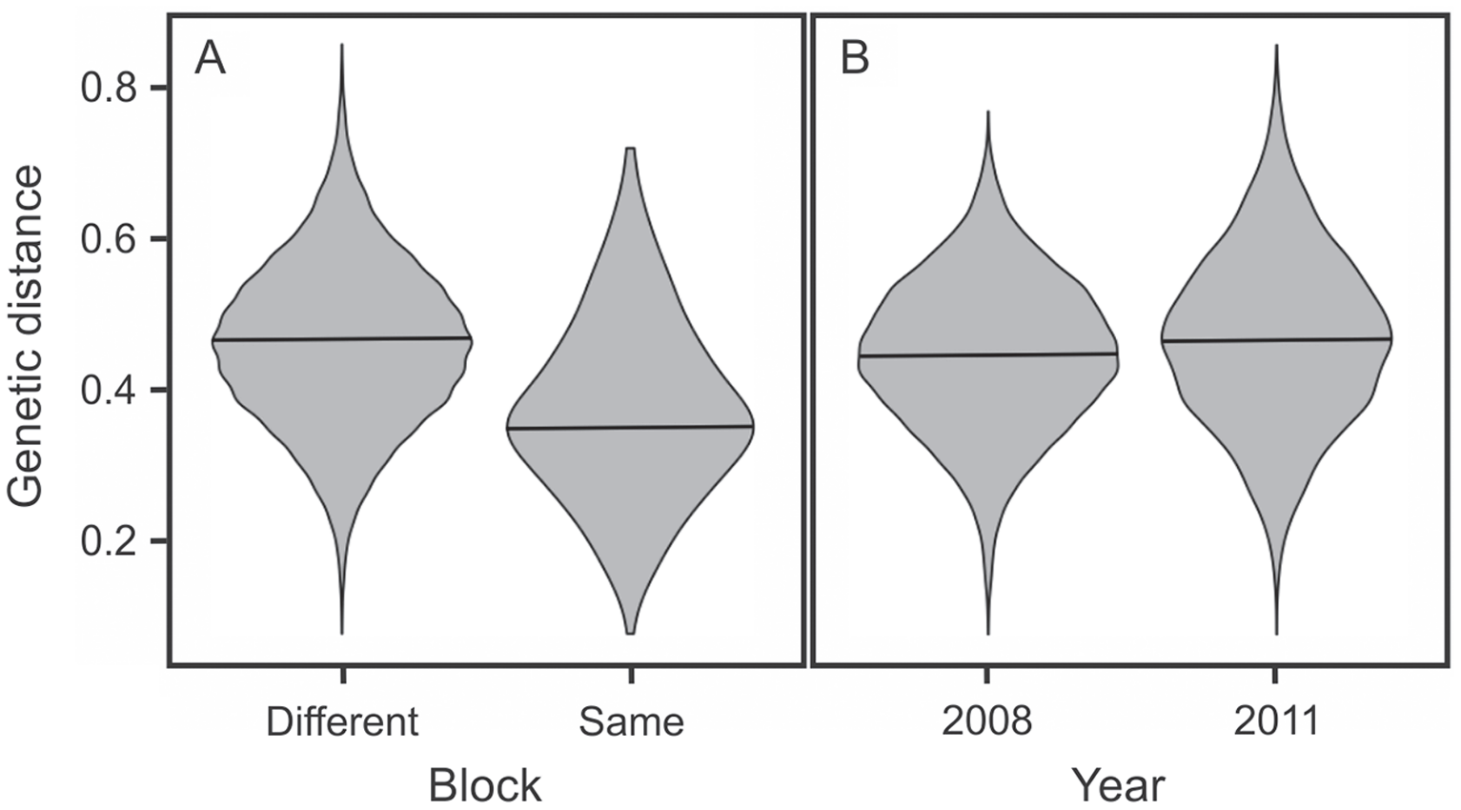
Violin plots displaying the distribution of genetic distance among pairs of samples. The effect of city block is large (panel A), whereas the sampling year has almost no effect (panel B).

The location of samples within or between city blocks has an important effect on the genetic dissimilarity among samples, which is visually evident in the violin plots (Fig. 3). The genetic dissimilarity between pairs of samples located on different blocks is substantially greater than the dissimilarity between pairs of samples located on the same block (Table 1). This effect remains statistically significant (p > 0.001) after controlling for the Euclidean distance between pairs of samples (S3 Fig.). The location of the samples (either within or between a city block) and the Euclidean distance among samples were statistically significant explanatory variables of the genetic dissimilarity among sampled insects (Table 1). In the reduced dataset that includes equally distanced pairs of individuals in the same or different blocks, no correlation was found between Euclidean distance and genetic distances (S4 Fig.; p > 0.05). A significant but very low correlation (S4 Fig.; R^2^ = 0.01, p < 0.001) was found in the complete dataset.

**Table 1:** Permutation test examining the influence of Euclidean distance, collection year, and city block, on genetic differences.

Factor	Regression coefficients	Statistical outcome
Euclidean distance	2.294e-5	*P* < 0.01
Year	2.181e-2	Not significant
City block	7.426e-2	*P* < 0.001

The regression coefficients and associated statistical outcomes are presented.

**Fig 4:**
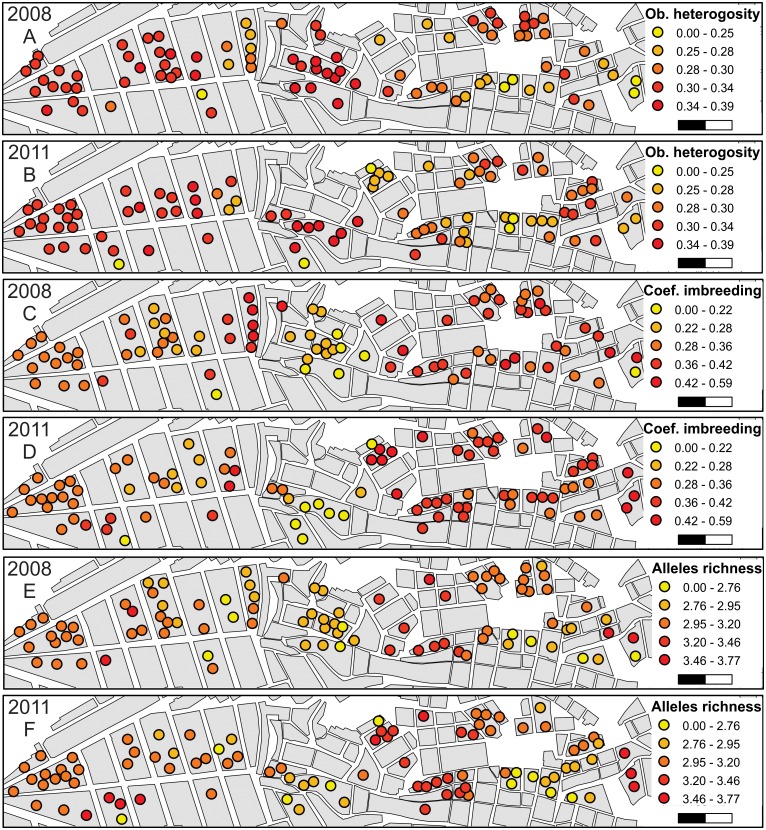
Point estimates of genetic diversity across the transect where each individual was considered the center of a neighborhood 225 meters in diameter. The inbreeding coefficient (A, B), observed heterozygosity (C, D), and allelic richness (E, F) were calculated for each collection year (2008: A, C, and E; 2011: B, D, and F) only when there were 10 or more individuals in each neighborhood. The scale bar represents 200 m. Data points have been relocated (jiggled) inside blocks to protect residents’ privacy.

**Fig 5:**
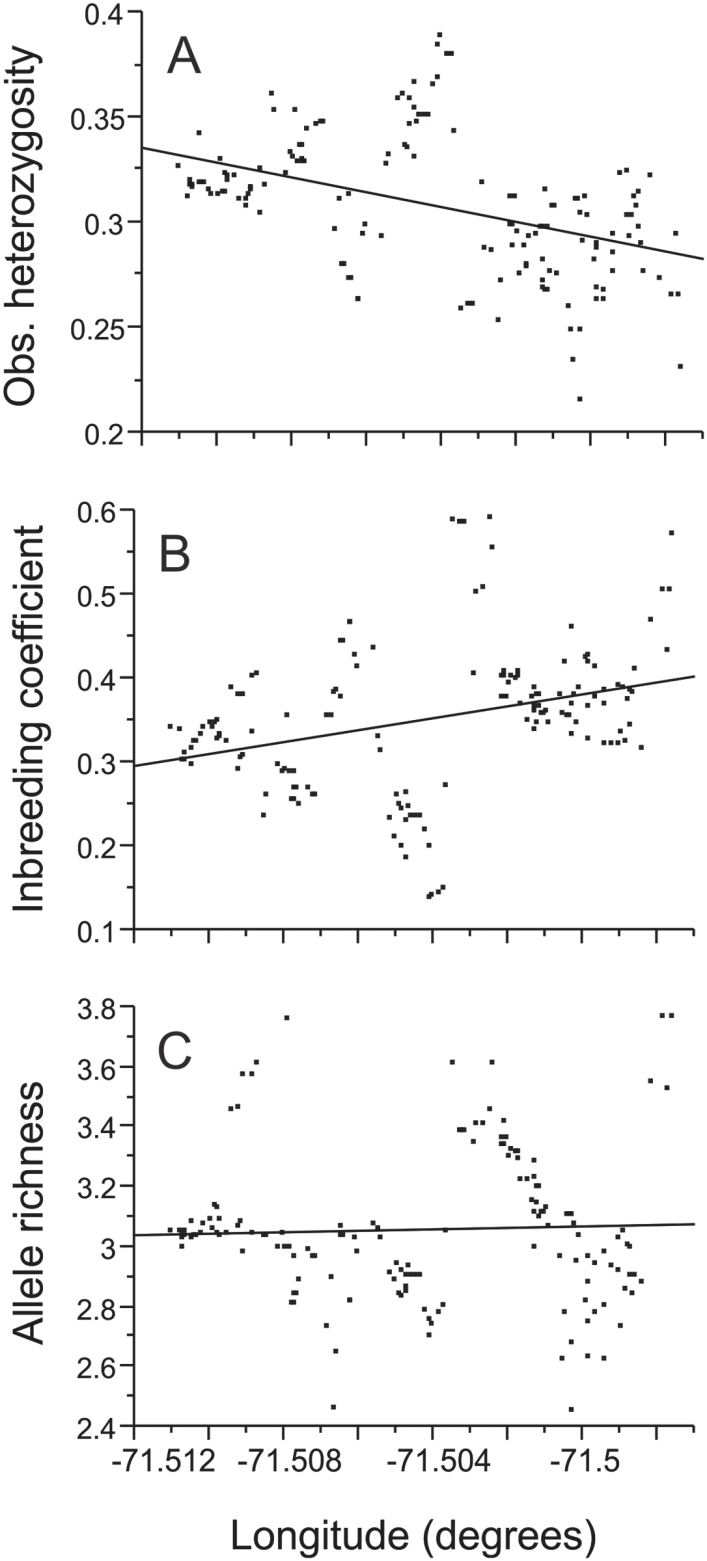
Longitudinal regression of the neighborhood indexes. Observed heterozygosity (R^2^ = 0.1962, F-ratio = 42.24, p < 0.0001; panel A), inbreeding coefficient, *F*_IS_ (R^2^ = 0.1064, F-ratio = 20.6, p < 0.0001; panel B), and allelic richness (R^2^ = 0.0016, F-ratio = 0.28, p = 0.6005; panel C).

The optimal radius distance for the neighborhood genetic diversity analyses was 225 m (S5 Fig.). The ages of the communities are related to the fine-scale genetic structure of the vector population, and statistical trends were detected from the west (older communities) to the east (newer communities) of the transect. The older parts of the transect had higher observed heterozygosity (Fig. 4A, 5A) and lower inbreeding coefficients (*F*_IS_; Fig. 4B, 5B). Allelic richness did not vary across the transect, (Fig. 4C, 5C), which is consistent with the observation that most common alleles (S2 Fig.) and STRUCTURE subpopulations (Fig. 2) can be found across the complete extension of the transect. The complete set of calculated indexes is presented in S2 Table. The patterns in local genetic diversity indexes are similar in the two collection years (Fig. 4).

## Discussion

The expansion of cities alters the environment and creates new habitats for urban species. In an expanding city, the genetic signature of an invading population is expected to be closely related to the history of urban development. However, urban environments contain many migratory barriers that may interfere with such expansion [30]. The data and analyses presented here confirm that city streets act as barriers to the dispersion of *T. infestans* and, consequently, that particular genotypes tend to be specific to a given city block.

The population genetic data from the study transect, which spans a gradient of urban development from established communities to recently-inhabited areas, describe a demographic history of *T. infestans* characterized by constant local dispersal and occasional long-distance migration events. Local dispersal events mainly involve households on the same city block, resulting in city blocks colonized by closely-related individuals (Fig. 3). Ambulatory movement, which occurs at all stages of the *T. infestans* life-cycle, is relatively rapid, as shown by sentinel habitat studies performed in this same system [31]. However, our data suggest that dispersal to neighboring houses across a city street is relatively rare. Multivariate regression models controlling for all potential confounding variables indicate that city streets remain strong environmental barriers to gene flow for *T. infestans* (Table 1, Figs. 3, S3, S4).

In several instances, closely related individuals can inhabit distant areas of the transect despite being absent from intervening blocks (Figs. 2, S2), suggesting occasional long distance migration events. These long distance migration events are relatively rare, as evidenced by the fact that most blocks are colonized by one single group of closely-related insects. Long distance dispersal may occur due to the natural flight capabilities of *T. infestans* or via human-mediated dispersal. Only *T. infestans* adults are capable of flight. In general, adults initiate flight only in response to severely limited resources [32], at which time they can cover distances of up to two kilometers [15,32,33], readily traversing several city blocks. Human-mediated dispersal can also promote dispersal events across several city blocks [34]. We cannot distinguish between aerial and human-mediated long distance dispersal in our transect, as both processes can result in the observed patterns.

Previous reports using presence-absence data have suggested that streets can act as barriers to dispersal for several insect species [35–38] including *T. infestans* [9]. However, these conclusions are potentially confounded by other factors such as clustering of environmental factors on either side of the putative barrier. Because of the danger posed by *T. infestans*, no mark-recapture studies that could elucidate their movement through the populated environment have been conducted. Our population genetics analysis allowed us to isolate the effects of streets on the dispersal of *T. infestans* from potential confounding explanations and thereby better describe the migratory and colonization processes of these insects in urban environments.

The genetic signatures typically associated with the recent introduction of a population, including limited heterozygosity and high inbreeding coefficients, were mostly associated with the newest section of the transect (Fig. 4, 5). These results support the temporal association between the time of first occurrence of *T. infestans* and the age of the communities in the transect, which was first described by Levy et al. (2014). These results confirm that *T. infestans* has been present for much longer in the older parts of the transect than in the newer parts. However, the analysis of the allelic richness adds a layer of complexity, as high levels of allelic richness are scattered throughout the transect (i.e., in both old and new communities). This mixed pattern can be explained by the connectivity of the transect to surrounding areas. These sections of great allelic richness could represent the location of the initial colonization or the location of subsequent contact with other *T. infestans* invasions.

The transition from new, recently urbanized communities to older, better established communities coincides with physical changes in housing structures; more specifically, land tenure tends to result in the acquisition of fully mortared walls and more domestic animals. Further changes can occur with increased capital, including the presence of fewer food animals and more companion animals [30]. These changes in host populations may have indirect effects on the population dynamics of *T. infestans*; however, these effects are very difficult to rigorously quantify. The genetic signatures detected in this study confirm that the demographic dynamics of *T. infestans* infestations are significantly affected by urbanization.

Our conclusions may guide the research and development of strategies to control the emergence and re-emergence of vector populations in urban environments. Isolated instances of *T. infestans* infestation or reinfestation are typically controlled by the application of insecticide around infested households in a ring-like fashion. Our results suggest that the initial application of insecticide should be focused on the city block where the infestation was first detected. Moreover, even though streets represent barriers for these insects, our results and those of previously published works [9] indicate that they are not impervious barriers and may be breached over time. Most importantly, our results call into question whether a purely spatial strategy of vector control such as the ring insecticide treatment can realistically lead to vector elimination, as this strategy can be overcome by repeated long-distance dispersal events. A better understanding of the social and migratory interactions of residents of infested houses may improve the long-term prospects of eliminating the vector from urban environments [11,15,30,34,39–42].

## Supporting Information

S1 Table List of primers used to amplify the microsatellites used in the study. This list includes the name of the primer, the source, the fluorescent dye used, forward and reverse sequences, and PCR cycling conditions.
(DOCX)

S2 Table Full list of the local genetic diversity indexes for each sample. The table details the ID of the sample, the size of the genetic neighborhood (n; number of samples inside the selected 225 m radius), the observed heterozygosity, the inbreeding coefficient (*F*_IS_), and the allelic richness. N/A indicates that the samples were not used because the neighborhood size was smaller than 10. Note that the geographical position has not been included to protect resident’s privacy.
(DOCX)

S1 Fig Determination of the optimal number of clusters using the *ΔK* method. This method is implemented by Structure Harvester. The selected number for *K* is four groups.
(DOCX)

S2 Fig Geographical distribution of the alleles from all of the studied loci. Individuals are represented by circles where each colored half circle represents one allele. Single color circles are homozygotes and two-color circles are heterozygotes. Additional circles over individuals highlight the presence of uncommon alleles in distant parts of the transect (one allele per locus). Alleles are presented in consecutive panels following the order reported in S1 Table. Data points have been relocated (jiggled) inside blocks to protect residents’ privacy.
(DOCX)

S3 Fig Block effect on genetic distance after controlling for Euclidean distances. The analysis is restricted to pairs of samples with matching distances (up to 1 m). The differences are significant (two-tailed paired t-test; t-ratio = 3.2691, *d.f*. = 132, p = 0.0014).
(DOCX)

S4 Fig Examination of the effect of Euclidean distances on genetic distances. In the restricted dataset, which only considers equally distanced pairs of individuals in the same (panel A; R^2^ = 0.0048; n = 133; p = 0.4277) or different city block (panel B; R^2^ = 0.0146; n = 133; p = 0.1657), Euclidean distances have no effect on genetic distances. In the complete dataset, Euclidean distances have small but significant effects on genetic distances (panel C; R^2^ = 0.01; n = 7,746; p = 0.0001).
(DOCX)

S5 Fig Selection of optimal radius (m) for calculating neighborhood indexes of genetic diversity using sGD. The selected distance (225 m) is indicated with an arrow. The optimal distance was considered to be the distance at which the increase in the number of valid samples levels off.
(DOCX)
