# Supplementary material for: Population Structure of the Chagas Disease Vector Triatoma infestans in an Urban Environment
Source: PLoS Negl Trop Dis. 2015 Feb 3;9(2):e0003425. doi: 10.1371/journal.pntd.0003425 (PMC4315598; doi:10.1371/journal.pntd.0003425)
Supplement: S1 Table — This list includes the name of the primer, the source, the fluorescent dye used, forward and reverse sequences, and PCR cycling conditions. (DOCX) [file pntd.0003425.s001.docx]

Supporting Table S1. List of primers used to amplify the microsatellites used in the study. This list includes the name of the primer, the source, the fluorescent dye used, forward and reverse sequences, and PCR cycling conditions.

| **Name** | **Source** | **DYE** | **Tagged 5' primer** | **3' Untagged primer** | **Annealing temperature (°C)** | **Annealing time (min)** |
| --- | --- | --- | --- | --- | --- | --- |
|  |  |  |  |  |  |  |
| TiA02 | Garcia et al., 2004 | PET | GGAAACTCATGTTATGGACACG | AAACCTTATTGTTAGTTCGTTTTGG | 55 | 1 |
| TiC02 | Garcia et al., 2004 | 6-FAM | CTCTGGGGATCATCGTTCTG | TTTAGGATTCATACCGCCTTT | 55 | 1 |
| TiC08 | Garcia et al., 2004 | 6-FAM | TTACTGCCACATTGCGTCAT | TCGTGATTGCAAGGAGGAAT | 52 | 1 |
| TiC09 | Garcia et al., 2004 | VIC | TTTGCCACATTTACCATTTCC | TCAAGAGAAGCCGTCCAACT | 52 | 1 |
| TiD09 | Garcia et al., 2004 | VIC | TGGACATAAGCCCCCTGTAA | GGATCCTACTGTGCGGATGT | 55 | 1 |
| TiE02 | Garcia et al., 2004 | 6-FAM | AGCACGGTTTGCAACTTTTC | TGTGGAATTGAAGGAGCACA | 55 | 1 |
| TiE12 | Garcia et al., 2004 | NED | CCTTTAATTTCCCTTTGCCATC | CCTACACGAAATGCCCAAGT | 55 | 0.5 |
| TiF03 | Garcia et al., 2004 | PET | AAAATGGCGGACAAACATTC | TCCTCAACACAAACACAAACC | 55 | 1 |
| TiM22 | Marcet et al., 2006 | NED | CGAGTCAAATTTTCCATGAGG | CCCATGGTGTTACCCAAAAC | 55 | 1 |
| TiM27 | Marcet et al., 2006 | PET | ATGAAGCCGAAACCACAAAG | GGGGAAGAGAATGCATTGAG | 55 | 0.5 |
| TiM3 | Marcet et al., 2006 | VIC | GCGGACTGAGAAAGGAACAC | TTCACCGCTCGTCTACACAC | 58 | 0.5 |
| TiM42 | Marcet et al., 2006 | NED | GACGCTCCAGCTATCGATTC | GGCCAATTGGTTTGGTAGTG | 55 | 1 |
| TiM5 | Marcet et al., 2006 | 6-FAM | ATGCTGATAGTCGCAACACG | TCGATCTTTTTCCCAAATCG | 55 | 1 |
|  |  |  |  |  |  |  |
