# Supplementary material for: Population Structure of the Chagas Disease Vector Triatoma infestans in an Urban Environment
Source: PLoS Negl Trop Dis. 2015 Feb 3;9(2):e0003425. doi: 10.1371/journal.pntd.0003425 (PMC4315598; doi:10.1371/journal.pntd.0003425)
Supplement: S2 Table — The table details the ID of the sample, the size of the genetic neighborhood (n; number of samples inside the selected 225 m radius), the observed heterozygosity, the inbreeding coefficient (F IS), and the allelic richness. N/A indicates that the samples were not used because the neighborhood size was smaller than 10. Note that the geographical position has not been included to protect resident’s privacy. (DOCX) [file pntd.0003425.s002.docx]

Supporting Table S2: Full list of the local genetic diversity indexes for each sample. The table details the ID of the sample, the size of the genetic neighborhood (n; number of samples inside the selected 225 m radius), the observed heterozygosity, the inbreeding coefficient (*F*_IS_, and the allelic richness. N/A indicates that the samples were not used because the neighborhood size was smaller than 10. Note that the geographical position has not been included to protect resident’s privacy.

| Sample ID | n | Obs. heterozygosity | Obs. heterozygosity (*F*_is_) | Allele Richness |
| --- | --- | --- | --- | --- |
| 1493 | 12 | 0.326923 | 0.342321 | 3.051282 |
| 44770 | 18 | 0.311966 | 0.337603 | 3.052887 |
| 1537 | 26 | 0.319527 | 0.302326 | 3.027844 |
| 2374 | 23 | 0.317726 | 0.30252 | 3.035869 |
| 44769 | 22 | 0.318182 | 0.3023 | 3.001594 |
| 2367 | 25 | 0.316923 | 0.310653 | 3.052744 |
| 44768 | 23 | 0.317726 | 0.30252 | 3.035869 |
| 46341 | 20 | 0.342308 | 0.297612 | 3.086568 |
| 1517 | 27 | 0.319088 | 0.315548 | 3.033784 |
| 47241 | 27 | 0.319088 | 0.315548 | 3.033784 |
| 44922 | 28 | 0.318681 | 0.325363 | 3.036031 |
| 1483 | 28 | 0.318681 | 0.325363 | 3.036031 |
| 1489 | 29 | 0.31565 | 0.333066 | 3.047047 |
| 1507 | 27 | 0.31339 | 0.341848 | 3.077466 |
| 46410 | 27 | 0.31339 | 0.341848 | 3.077466 |
| 1490 | 30 | 0.312821 | 0.346811 | 3.09458 |
| 1511 | 17 | 0.330317 | 0.340486 | 3.064349 |
| 1532 | 32 | 0.314904 | 0.34784 | 3.139257 |
| 39991 | 32 | 0.314904 | 0.34784 | 3.139257 |
| 1485 | 29 | 0.323607 | 0.32736 | 3.045235 |
| 39908 | 29 | 0.323607 | 0.32736 | 3.045235 |
| 1523 | 33 | 0.314685 | 0.348662 | 3.133664 |
| 39857 | 33 | 0.314685 | 0.348662 | 3.133664 |
| 40774 | 31 | 0.322581 | 0.3303 | 3.090277 |
| 1591 | 26 | 0.319527 | 0.332262 | 3.034393 |
| 40138 | 26 | 0.319527 | 0.332262 | 3.034393 |
| 47184 | 26 | 0.322485 | 0.324576 | 3.047113 |
| 1530 | 23 | 0.311037 | 0.38898 | 3.46156 |
| 46408 | 13 | 0.307692 | 0.291312 | 3.071006 |
| 1531 | 23 | 0.311037 | 0.381499 | 3.464515 |
| 1657 | 14 | 0.313187 | 0.305204 | 3.081995 |
| 1465 | 21 | 0.315018 | 0.381072 | 3.571361 |
| 1634 | 17 | 0.316742 | 0.308855 | 2.98016 |
| 44946 | 21 | 0.326007 | 0.336811 | 3.044692 |
| 40226 | 24 | 0.304487 | 0.402516 | 3.572276 |
| 44901 | 23 | 0.317726 | 0.404219 | 3.611673 |
| 1630 | 20 | 0.361538 | 0.235936 | 3.040632 |
| 1516 | N/A | N/A | N/A | N/A |
| 46767 | 22 | 0.353147 | 0.260073 | 3.036969 |
| 1429 | 25 | 0.323077 | 0.29805 | 3.002204 |
| 40311 | 24 | 0.333333 | 0.287671 | 3.042626 |
| 40306 | 23 | 0.331104 | 0.291246 | 2.995525 |
| 1629 | 15 | 0.353846 | 0.354278 | 3.763314 |
| 1414 | 22 | 0.328671 | 0.289033 | 3.00125 |
| 41135 | 22 | 0.328671 | 0.289033 | 3.00125 |
| 1434 | 21 | 0.336996 | 0.254759 | 2.809779 |
| 41759 | 21 | 0.336996 | 0.254759 | 2.809779 |
| 47092 | 21 | 0.32967 | 0.290221 | 2.971159 |
| 46632 | 22 | 0.328671 | 0.268889 | 2.843093 |
| 1439 | 22 | 0.328671 | 0.268889 | 2.843093 |
| 40366 | 22 | 0.328671 | 0.268889 | 2.843093 |
| 45811 | 23 | 0.344482 | 0.24942 | 2.890653 |
| 1457 | 24 | 0.346154 | 0.268551 | 2.994617 |
| 46933 | 24 | 0.346154 | 0.268551 | 2.994617 |
| 1424 | 25 | 0.347692 | 0.262242 | 2.970439 |
| 47159 | 25 | 0.347692 | 0.262242 | 2.970439 |
| 1405 | 25 | 0.347692 | 0.262242 | 2.970439 |
| 1526 | 22 | 0.297203 | 0.355014 | 2.734048 |
| 41271 | N/A | N/A | N/A | N/A |
| 1574 | 24 | 0.310897 | 0.354549 | 2.896963 |
| 45955 | 11 | 0.27972 | 0.382239 | 2.461538 |
| 40544 | 14 | 0.28022 | 0.385257 | 2.650888 |
| 1419 | 13 | 0.313609 | 0.376776 | 3.071006 |
| 1491 | 20 | 0.273077 | 0.445768 | 3.038219 |
| 40400 | 13 | 0.313609 | 0.376776 | 3.071006 |
| 1515 | 20 | 0.273077 | 0.445768 | 3.038219 |
| 45969 | 14 | 0.263736 | 0.465296 | 2.818259 |
| 45968 | 14 | 0.263736 | 0.465296 | 2.818259 |
| 45967 | 14 | 0.263736 | 0.465296 | 2.818259 |
| 45964 | 18 | 0.294872 | 0.428223 | 3.027478 |
| 45963 | 17 | 0.298643 | 0.414147 | 2.983597 |
| 44786 | 11 | 0.293706 | 0.436997 | 3.076923 |
| 1879 | 23 | 0.327759 | 0.330019 | 3.064645 |
| 11387 | 22 | 0.332168 | 0.312898 | 3.03248 |
| 1897 | 18 | 0.358974 | 0.232464 | 2.916502 |
| 1899 | 20 | 0.361538 | 0.212001 | 2.89372 |
| 1528 | N/A | N/A | N/A | N/A |
| 44763 | 21 | 0.336996 | 0.260896 | 2.942354 |
| 45064 | 11 | 0.335664 | 0.248826 | 2.846154 |
| 1553 | 15 | 0.358974 | 0.199346 | 2.836292 |
| 44787 | 22 | 0.346154 | 0.245235 | 2.923864 |
| 40781 | 23 | 0.354515 | 0.230744 | 2.90869 |
| 1896 | 21 | 0.3663 | 0.18633 | 2.853616 |
| 40817 | 21 | 0.3663 | 0.18633 | 2.853616 |
| 41286 | 13 | 0.331361 | 0.265172 | 2.869822 |
| 46580 | 23 | 0.347826 | 0.248357 | 2.937923 |
| 41377 | 23 | 0.351171 | 0.235353 | 2.907069 |
| 41762 | 23 | 0.351171 | 0.235353 | 2.907069 |
| 41280 | 23 | 0.351171 | 0.235353 | 2.907069 |
| 44658 | 20 | 0.365385 | 0.219965 | 2.786584 |
| 1863 | 19 | 0.384615 | 0.138973 | 2.700258 |
| 45461 | 20 | 0.369231 | 0.19877 | 2.755766 |
| 41068 | 19 | 0.388664 | 0.14094 | 2.740973 |
| 1996 | 16 | 0.379808 | 0.143167 | 2.778001 |
| 44415 | 17 | 0.38009 | 0.150174 | 2.805952 |
| 13573 | N/A | N/A | N/A | N/A |
| 13545 | 15 | 0.34359 | 0.271173 | 3.056072 |
| 13560 | 11 | 0.258741 | 0.588203 | 3.615385 |
| 13552 | 10 | 0.261538 | 0.585647 | 3.384615 |
| 46611 | 10 | 0.261538 | 0.585647 | 3.384615 |
| 13579 | 10 | 0.261538 | 0.585647 | 3.384615 |
| 13565 | 10 | 0.261538 | 0.585647 | 3.384615 |
| 12964 | N/A | N/A | N/A | N/A |
| 41376 | 14 | 0.318681 | 0.404423 | 3.348267 |
| 13574 | 19 | 0.287449 | 0.503978 | 3.407808 |
| 44757 | 18 | 0.286325 | 0.509158 | 3.409986 |
| 46595 | 10 | 0.253846 | 0.591753 | 3.461538 |
| 14556 | 11 | 0.272727 | 0.554795 | 3.615385 |
| 12687 | 18 | 0.299145 | 0.404106 | 3.338632 |
| 39359 | 18 | 0.299145 | 0.404106 | 3.338632 |
| 12376 | 17 | 0.312217 | 0.376447 | 3.363775 |
| 39336 | 18 | 0.299145 | 0.404106 | 3.338632 |
| 12902 | 21 | 0.289377 | 0.409235 | 3.415748 |
| 12380 | 17 | 0.312217 | 0.376447 | 3.363775 |
| 12418 | 19 | 0.295547 | 0.395028 | 3.303232 |
| 43983 | 17 | 0.276018 | 0.403605 | 3.323877 |
| 39490 | 20 | 0.288462 | 0.399621 | 3.32031 |
| 14567 | 19 | 0.279352 | 0.403745 | 3.297901 |
| 13138 | 22 | 0.27972 | 0.408138 | 3.31793 |
| 14558 | 22 | 0.293706 | 0.368647 | 3.223714 |
| 45078 | 17 | 0.294118 | 0.349797 | 3.224765 |
| 14585 | 23 | 0.297659 | 0.377127 | 3.156864 |
| 12684 | 18 | 0.282051 | 0.361956 | 3.228526 |
| 14595 | 23 | 0.297659 | 0.366753 | 3.115767 |
| 45106 | 23 | 0.297659 | 0.366753 | 3.115767 |
| 12442 | 26 | 0.272189 | 0.339175 | 2.996784 |
| 12700 | 22 | 0.269231 | 0.390157 | 3.288847 |
| 14578 | 19 | 0.315789 | 0.34728 | 3.145282 |
| 45096 | 19 | 0.315789 | 0.34728 | 3.145282 |
| 45931 | 21 | 0.267399 | 0.380042 | 3.198466 |
| 12631 | 21 | 0.267399 | 0.380042 | 3.198466 |
| 14602 | 23 | 0.297659 | 0.366343 | 3.099031 |
| 44709 | 23 | 0.297659 | 0.366343 | 3.099031 |
| 39801 | 25 | 0.276923 | 0.358289 | 3.11182 |
| 46625 | 20 | 0.307692 | 0.35892 | 3.115412 |
| 14591 | 22 | 0.307692 | 0.360885 | 3.130869 |
| 46593 | 22 | 0.307692 | 0.360885 | 3.130869 |
| 39580 | 24 | 0.275641 | 0.348592 | 3.069291 |
| 12769 | 21 | 0.260073 | 0.381533 | 2.969537 |
| 13523 | 17 | 0.248869 | 0.35696 | 2.629003 |
| 39784 | 20 | 0.234615 | 0.420065 | 2.784711 |
| 45109 | 20 | 0.311538 | 0.354852 | 3.111189 |
| 14610 | 20 | 0.311538 | 0.354852 | 3.111189 |
| 45116 | 20 | 0.311538 | 0.354852 | 3.111189 |
| 12694 | 17 | 0.248869 | 0.333333 | 2.458058 |
| 45102 | 24 | 0.304487 | 0.369772 | 3.10804 |
| 39609 | 20 | 0.215385 | 0.460856 | 2.679063 |
| 44705 | 28 | 0.291209 | 0.380519 | 3.07549 |
| 44712 | 17 | 0.312217 | 0.347518 | 2.951923 |
| 14593 | 17 | 0.303167 | 0.387778 | 3.034285 |
| 15344 | 15 | 0.282051 | 0.424514 | 2.818259 |
| 12982 | 21 | 0.263736 | 0.366755 | 2.753564 |
| 45094 | 17 | 0.289593 | 0.427773 | 2.885442 |
| 39787 | 20 | 0.269231 | 0.328622 | 2.629518 |
| 45093 | 23 | 0.287625 | 0.418472 | 2.965576 |
| 14736 | 21 | 0.267399 | 0.376601 | 2.780881 |
| 41372 | 26 | 0.263314 | 0.413548 | 2.943298 |
| 44861 | 29 | 0.29443 | 0.386377 | 2.986889 |
| 14922 | 22 | 0.276224 | 0.370518 | 2.808112 |
| 14754 | 17 | 0.285068 | 0.321897 | 2.625827 |
| 14726 | 25 | 0.323077 | 0.321851 | 2.933226 |
| 43120 | 25 | 0.323077 | 0.321851 | 2.933226 |
| 43488 | 25 | 0.323077 | 0.321851 | 2.933226 |
| 14706 | 19 | 0.303644 | 0.392166 | 3.03183 |
| 14776 | 23 | 0.324415 | 0.320923 | 2.920287 |
| 14737 | 16 | 0.293269 | 0.337437 | 2.733411 |
| 42553 | 16 | 0.293269 | 0.337437 | 2.733411 |
| 42164 | 20 | 0.303846 | 0.388595 | 3.049774 |
| 44858 | 19 | 0.311741 | 0.325712 | 2.859976 |
| 14704 | 16 | 0.307692 | 0.375813 | 3.007396 |
| 14734 | 16 | 0.298077 | 0.386139 | 2.995773 |
| 14854 | 23 | 0.314381 | 0.344948 | 2.906059 |
| 14783 | 17 | 0.289593 | 0.384615 | 2.841585 |
| 42160 | 20 | 0.276923 | 0.412119 | 2.904139 |
| 14702 | 21 | 0.322344 | 0.317565 | 2.885951 |
| 42147 | 20 | 0.273077 | 0.470357 | 3.5545 |
| 14861 | 11 | 0.265734 | 0.505208 | 3.769231 |
| 14939 | 12 | 0.294872 | 0.434321 | 3.525641 |
| 14771 | 11 | 0.265734 | 0.505208 | 3.769231 |
| 42792 | 10 | 0.230769 | 0.572785 | 3.769231 |
| 43454 | N/A | N/A | N/A | N/A |
| 45341 | N/A | N/A | N/A | N/A |
