# Supplementary material for: Population Structure of the Chagas Disease Vector Triatoma infestans in an Urban Environment
Source: PLoS Negl Trop Dis. 2015 Feb 3;9(2):e0003425. doi: 10.1371/journal.pntd.0003425 (PMC4315598; doi:10.1371/journal.pntd.0003425)
Supplement: S1 Fig — This method is implemented by Structure Harvester. The selected number for K is four groups. (DOCX) [file pntd.0003425.s003.docx]

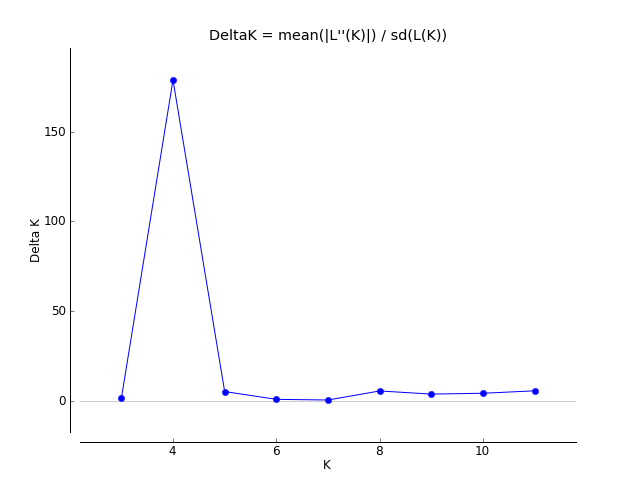


Supporting Figure S1. Determination of the optimal number of clusters using the ΔK method. This method is implemented by Structure Harvester. The selected number for K is four groups.
