# Supplementary material for: Population Structure of the Chagas Disease Vector Triatoma infestans in an Urban Environment
Source: PLoS Negl Trop Dis. 2015 Feb 3;9(2):e0003425. doi: 10.1371/journal.pntd.0003425 (PMC4315598; doi:10.1371/journal.pntd.0003425)
Supplement: S2 Fig — Individuals are represented by circles where each colored half circle represents one allele. Single color circles are homozygotes and two-color circles are heterozygotes. Additional circles over individuals highlight the presence of uncommon alleles in distant parts of the transect (one allele per locus). Alleles are presented in consecutive panels following the order reported in S1 Table. Data points have been relocated (jiggled) inside blocks to protect residents’ privacy. (DOCX) [file pntd.0003425.s004.docx]

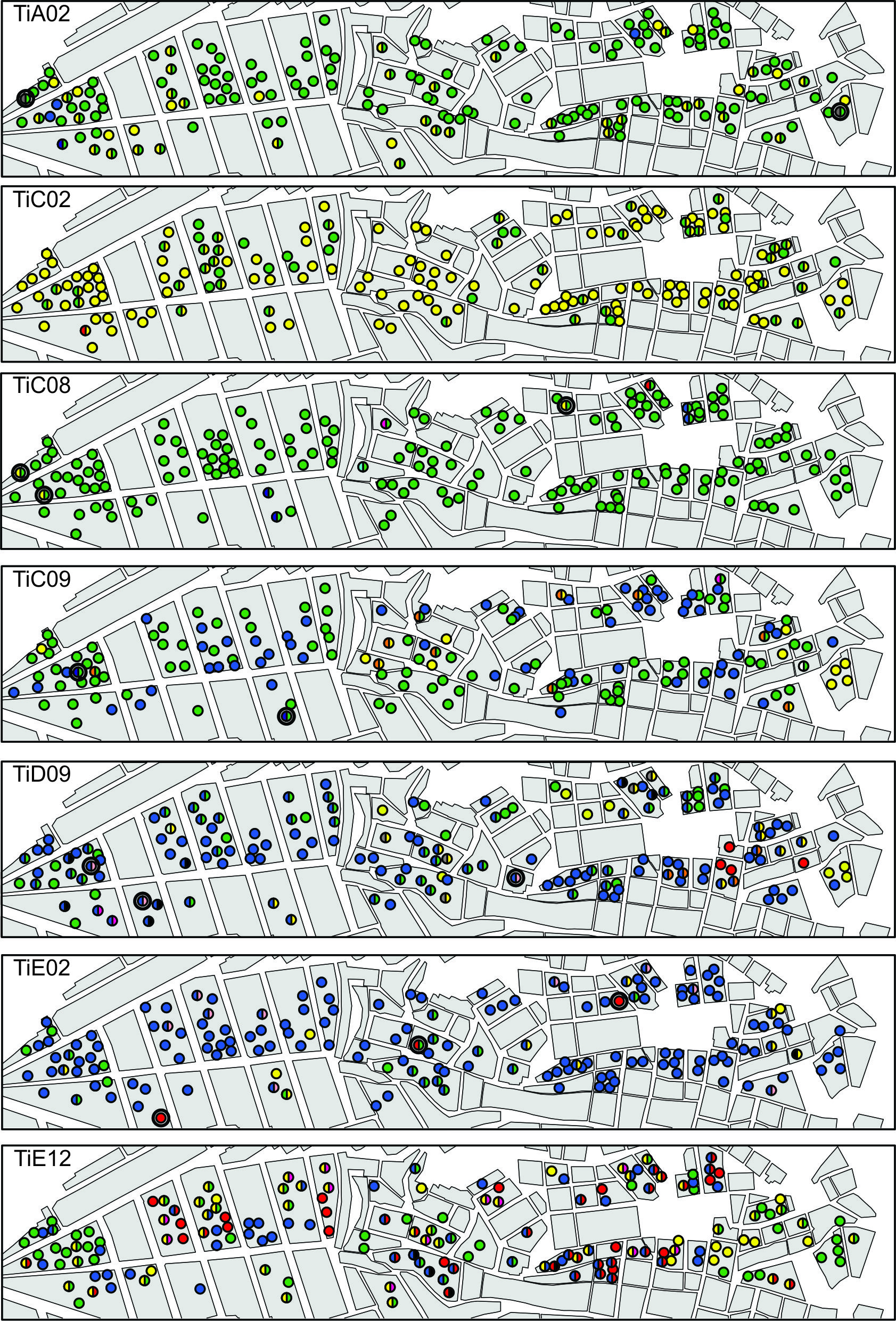


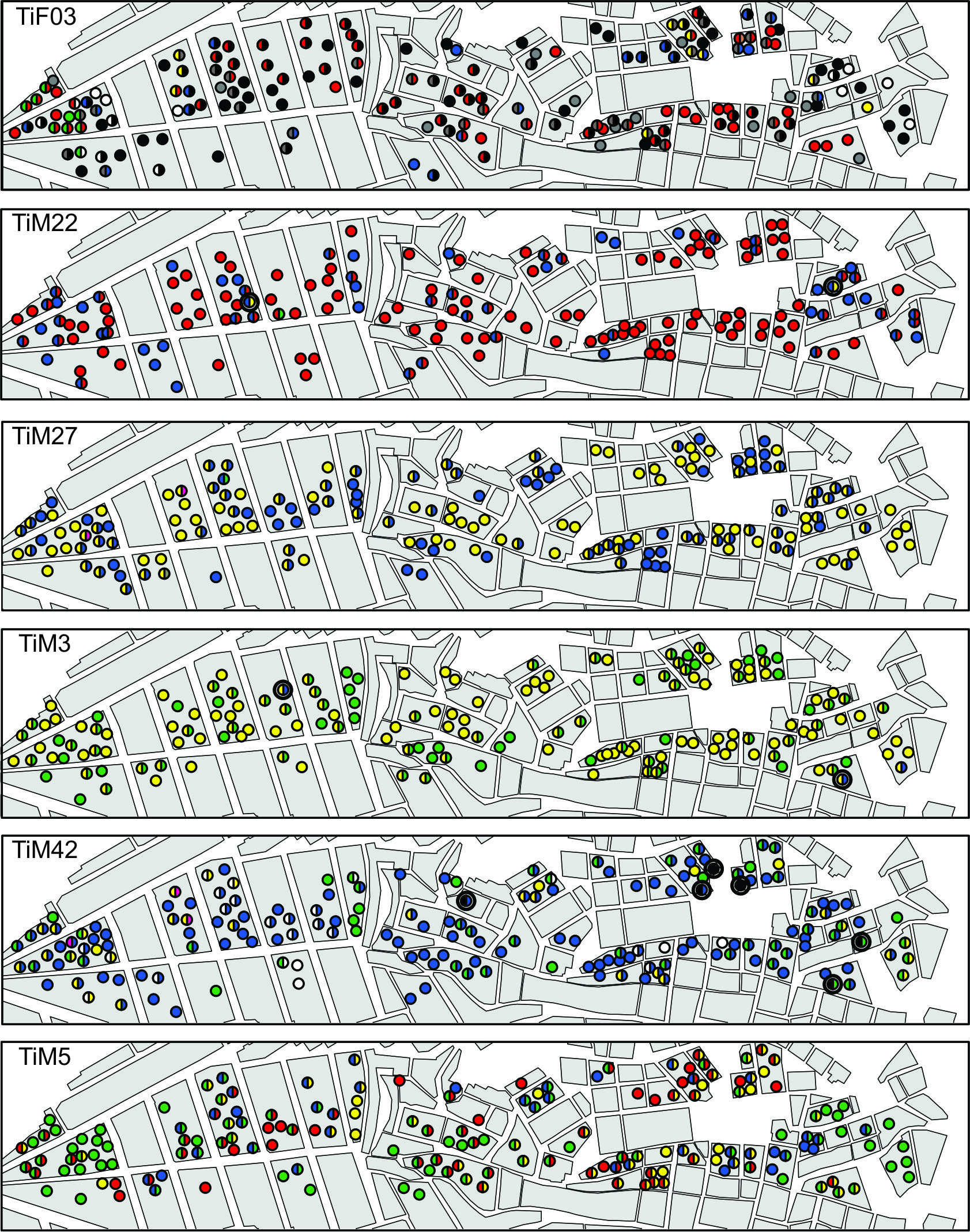


Supplemental Figure S2. Geographical distribution of the alleles from all of the studied loci. Individuals are represented by circles where each colored half circle represents one allele. Single color circles are homozygotes and two-color circles are heterozygotes. Additional circles over individuals highlight the presence of uncommon alleles in distant parts of the transect (one allele per locus). Alleles are presented in consecutive panels following the order reported in Table S1. Data points have been relocated (jiggled) inside blocks to protect residents’ privacy.
