# Supplementary material for: Population Structure of the Chagas Disease Vector Triatoma infestans in an Urban Environment
Source: PLoS Negl Trop Dis. 2015 Feb 3;9(2):e0003425. doi: 10.1371/journal.pntd.0003425 (PMC4315598; doi:10.1371/journal.pntd.0003425)
Supplement: S3 Fig — The analysis is restricted to pairs of samples with matching distances (up to 1 m). The differences are significant (two-tailed paired t-test; t-ratio = 3.2691, d.f. = 132, p = 0.0014). (DOCX) [file pntd.0003425.s005.docx]

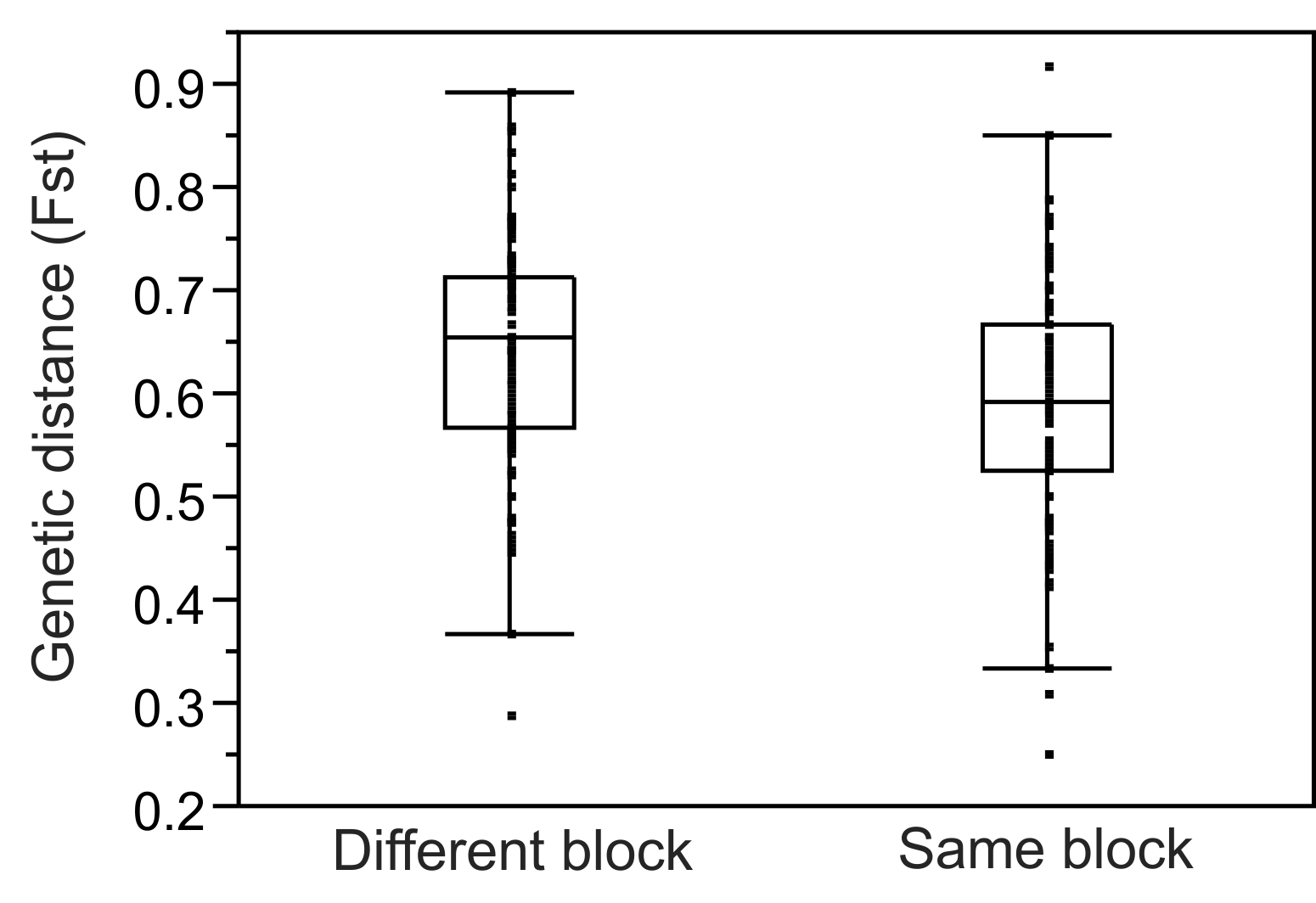


Supplemental Figure S3. Block effect on genetic distance after controlling for Euclidean distances. The analysis is restricted to pairs of samples with matching distances (up to 1 m). The differences are significant (two-tailed paired t-test; t-ratio = 3.2691, *d.f.* = 132, p = 0.0014).
