# Supplementary material for: Population Structure of the Chagas Disease Vector Triatoma infestans in an Urban Environment
Source: PLoS Negl Trop Dis. 2015 Feb 3;9(2):e0003425. doi: 10.1371/journal.pntd.0003425 (PMC4315598; doi:10.1371/journal.pntd.0003425)
Supplement: S4 Fig — In the restricted dataset, which only considers equally distanced pairs of individuals in the same (panel A; R2 = 0.0048; n = 133; p = 0.4277) or different city block (panel B; R2 = 0.0146; n = 133; p = 0.1657), Euclidean distances have no effect on genetic distances. In the complete dataset, Euclidean distances have small but significant effects on genetic distances (panel C; R2 = 0.01; n = 7,746; p = 0.0001). (DOCX) [file pntd.0003425.s006.docx]

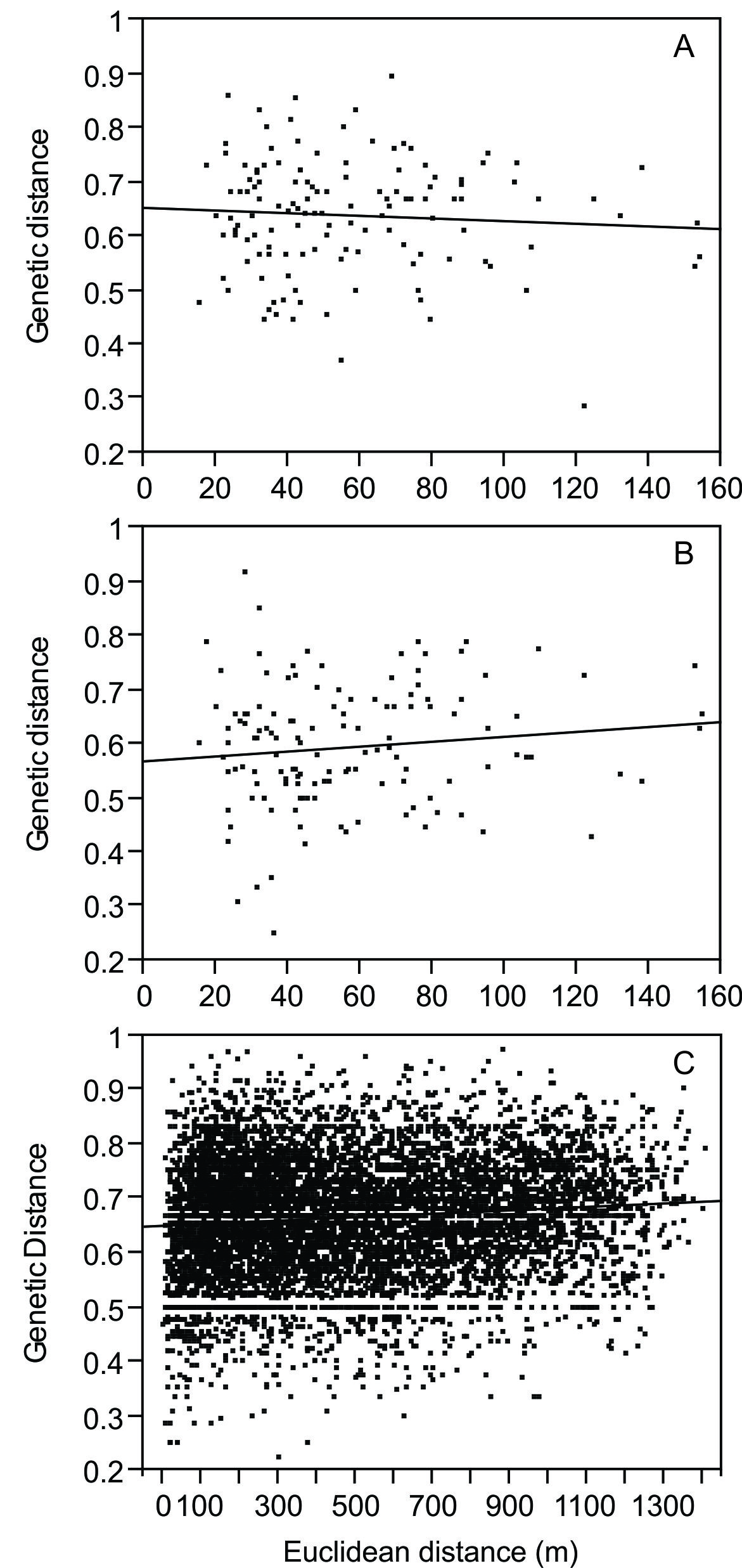


Supplemental Figure S4. Examination of the effect of Euclidean distances on genetic distances. In the restricted dataset, which only considers equally distanced pairs of individuals in the same (panel A; R^2^ = 0.0048; n = 133; p = 0.4277) or different city block (panel B; R^2^ = 0.0146; n = 133; p = 0.1657), Euclidean distances have no effect on genetic distances. In the complete dataset, Euclidean distances have small but significant effects on genetic distances (panel C; R^2^ = 0.01; n = 7,746; p = 0.0001).
