# Supplementary material for: Population Structure of the Chagas Disease Vector Triatoma infestans in an Urban Environment
Source: PLoS Negl Trop Dis. 2015 Feb 3;9(2):e0003425. doi: 10.1371/journal.pntd.0003425 (PMC4315598; doi:10.1371/journal.pntd.0003425)
Supplement: S5 Fig — The selected distance (225 m) is indicated with an arrow. The optimal distance was considered to be the distance at which the increase in the number of valid samples levels off. (DOCX) [file pntd.0003425.s007.docx]

Supplemental Figure S5. Selection of optimum radius (m) for calculating neighborhood indexes of genetic diversity using sGD. The selected distance (225 m) is indicated with an arrow. The optimal distance was defined as the distance where the number of valid samples is approaching a plateau.
